# Supplementary material for: A stromal Integrated Stress Response activates perivascular cancer-associated fibroblasts to drive angiogenesis and tumour progression
Source: Nat Cell Biol. 2022 Jun 2;24(6):940–53. doi: 10.1038/s41556-022-00918-8 (PMC9203279; doi:10.1038/s41556-022-00918-8)
Supplement: Supplementary file 1 — Supplementary Figs. 1 and 2. [file 41556_2022_918_MOESM1_ESM.pdf]

nature portfolio

https://doi.org/10.1038/s41556-022-00918-8

Supplementary information

A stromal Integrated Stress Response activates perivascular cancer-associated fibroblasts to drive angiogenesis and tumour progression

In the format provided by the authors and unedited

Gating strategy for live/dead cells

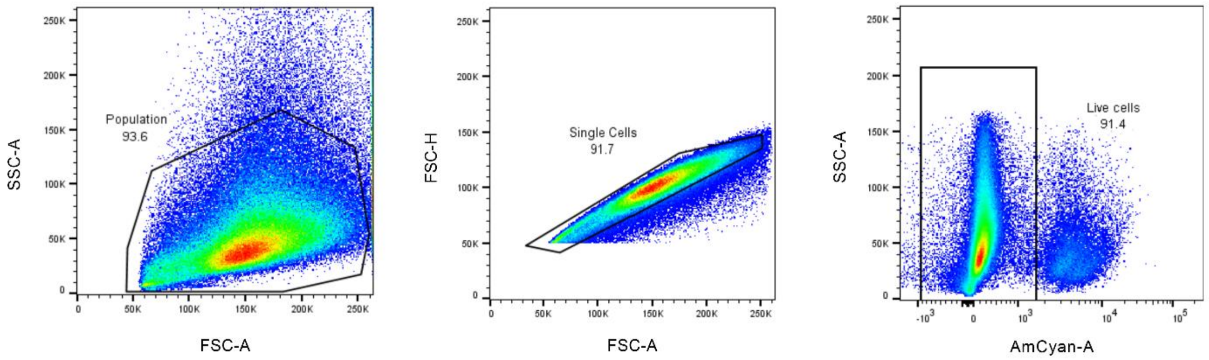

Supplementary Figure 1. FACS gating strategy for live/dead cells.
Forward scatter (FSC-A) versus side scatter (SSC-A) gating were used for the selection of population. FSC-A versus FSC-H gating were used for the selection of single cells only. AmCyan-A versus SSC-A were used for the selection of live/dead cells.

Gating strategy for CD31+ cells

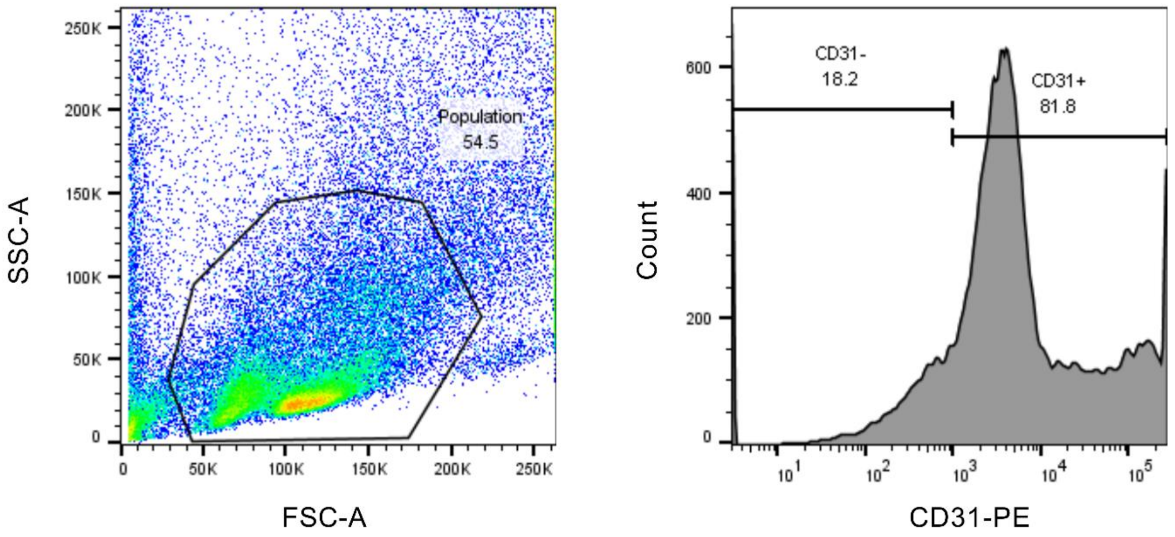

Supplementary Figure 2. Gating strategy for CD31+ cells.
Forward scatter (FSC-A) versus side scatter (SSC-A) gating were used for the selection of population. PE-A versus count were used for the selection of CD31+/CD31- cells.
