# Supplementary material for: A stromal Integrated Stress Response activates perivascular cancer-associated fibroblasts to drive angiogenesis and tumour progression
Source: Nat Cell Biol. 2022 Jun 2;24(6):940–53. doi: 10.1038/s41556-022-00918-8 (PMC9203279; doi:10.1038/s41556-022-00918-8)
Supplement: Supplementary file 2 — Reporting Summary [file 41556_2022_918_MOESM2_ESM.pdf]

nature portfolio

Corresponding author(s): Constantinos Koumenis

Last updated by author(s): Mar 25, 2022

Reporting Summary

Nature Portfolio wishes to improve the reproducibility of the work that we publish. This form provides structure for consistency and transparency in reporting. For further information on Nature Portfolio policies, see our Editorial Policies and the Editorial Policy Checklist.

Statistics

For all statistical analyses, confirm that the following items are present in the figure legend, table legend, main text, or Methods section.

n/a	Confirmed	
☐	☒	The exact sample size (*n*) for each experimental group/condition, given as a discrete number and unit of measurement
☐	☒	A statement on whether measurements were taken from distinct samples or whether the same sample was measured repeatedly
☐	☒	The statistical test(s) used AND whether they are one- or two-sided<br>*Only common tests should be described solely by name; describe more complex techniques in the Methods section.*
☒	☐	A description of all covariates tested
☒	☐	A description of any assumptions or corrections, such as tests of normality and adjustment for multiple comparisons
☐	☒	A full description of the statistical parameters including central tendency (e.g. means) or other basic estimates (e.g. regression coefficient) AND variation (e.g. standard deviation) or associated estimates of uncertainty (e.g. confidence intervals)
☐	☒	For null hypothesis testing, the test statistic (e.g. *F*, *t*, *r*) with confidence intervals, effect sizes, degrees of freedom and *P* value noted<br>*Give P values as exact values whenever suitable.*
☒	☐	For Bayesian analysis, information on the choice of priors and Markov chain Monte Carlo settings
☒	☐	For hierarchical and complex designs, identification of the appropriate level for tests and full reporting of outcomes
☐	☒	Estimates of effect sizes (e.g. Cohen's *d*, Pearson's *r*), indicating how they were calculated

Our web collection on statistics for biologists contains articles on many of the points above.

Software and code

Policy information about availability of computer code

Data collection	QuantStudio 6 Flex, ChemiDoc, MAVEN, Cell Ranger V3.0.1, R package ggplot2, OncoLnc, BD FFACSCanto II, NIS Elements v.4.60.00, Zen 3.1
Data analysis	Excel 365, GraphPad Prism version 8, Image J (FIJI), ChemiDoc, MAVEN, Cell Ranger V3.0.1, R package ggplot2, OncoLnc, RSAT, FlowJo v.10, NIS Elements v.4.60.00, Zen 3.1, FastQC, Cutadapt, Imaris 9.8.1

For manuscripts utilizing custom algorithms or software that are central to the research but not yet described in published literature, software must be made available to editors and reviewers. We strongly encourage code deposition in a community repository (e.g. GitHub). See the Nature Portfolio guidelines for submitting code & software for further information.

Data

Policy information about availability of data

All manuscripts must include a data availability statement. This statement should provide the following information, where applicable:

- Accession codes, unique identifiers, or web links for publicly available datasets
- A description of any restrictions on data availability
- For clinical datasets or third party data, please ensure that the statement adheres to our policy

scRNA–seq and microarray data that support the findings of this study have been deposited in the Gene Expression Omnibus (GEO) under accession codes GSE159996 and GSE159020. The human skin cutaneous melanoma and pancreatic adenocarcinoma data were derived from Broad GDAC Firehose: https://gdac.broadinstitute.org/. Publicly available ChIP-Seq datasets used in this study were retrieved from GEO repository and can be found under the accession codes GSM873426 and GSM873427.
All other data and scripts supporting the findings of this study are available from the corresponding author on reasonable request.

Field-specific reporting

Please select the one below that is the best fit for your research. If you are not sure, read the appropriate sections before making your selection.

☒ Life sciences ☐ Behavioural & social sciences ☐ Ecological, evolutionary & environmental sciences

For a reference copy of the document with all sections, see nature.com/documents/nr-reporting-summary-flat.pdf

Life sciences study design

All studies must disclose on these points even when the disclosure is negative.

Sample size	Statistical method was not used to predetermine sample size. We determined the sample size based on previous publications of our group and others and our experience.
Data exclusions	There was no inclusion/exclusion criteria for samples as no sample results were excluded
Replication	All experiments were performed at 1-3 times unless noted in the legend. All attempts at replication were successful.
Randomization	There was no randomization of the mice since post tamoxifen treatment we had to follow the excision of ATF4. No specific treatment was given to the mice so randomization was not possible.
Blinding	Tumor growth measurements in mice, quantification of immunofluorescence images and two photon microscopy, were blinded by use of coded subjects (mice).

Reporting for specific materials, systems and methods

We require information from authors about some types of materials, experimental systems and methods used in many studies. Here, indicate whether each material, system or method listed is relevant to your study. If you are not sure if a list item applies to your research, read the appropriate section before selecting a response.

Materials & experimental systems

n/a	Involved in the study	
☐	☒	Antibodies
☐	☒	Eukaryotic cell lines
☒	☐	Palaeontology and archaeology
☐	☒	Animals and other organisms
☐	☒	Human research participants
☒	☐	Clinical data
☒	☐	Dual use research of concern

Methods

n/a	Involved in the study	
☒	☐	ChIP-seq
☐	☒	Flow cytometry
☒	☐	MRI-based neuroimaging

Antibodies

Antibodies used	Rabbit monoclonal anti-ATF4 (Clone: D4B8, Cat#11815, RRID:AB_2616025), Rabbit monoclonal anti-PERK (Clone: C33E10, Cat#3192, RRID:AB_2095847), Rabbit monoclonal anti-p-eIF2a (Clone: D9G8, Cat#3398, RRID:AB_2096481), Rabbit polyclonal anti-eIF2a (Cat#9722, RRID:AB_2230924), Rabbit monoclonal anti-p-SMAD3 (Clone: C25A9, Cat#9520, RRID:AB_2193207), Rabbit monoclonal anti-SMAD2/3 (Clone: D7G7, Cat#8685, RRID:AB_10889933), Mouse monoclonal anti-b-actin (Clone: 8H10D10, Cat#3700, RRID:AB_2242334), Rabbit polyclonal anti-b-tubulin (Cat#2146, RRID:AB_2210545), Normal Rabbit IgG (Cat#2729S, RRID:AB_1031062) were purchased from Cell Signaling Technology. Rabbit polyclonal anti-Collagen Type I (Cat#AB765P, RRID:AB_92259), Mouse monoclonal anti-Acta2 (aSMA), Cy3 (Cat#C6198, RRID:AB_476856), Mouse monoclonal anti- Acta2, FITC (Clone: 1A4, Cat#F3777, RRID:AB_476977) were purchased from Millipore-Sigma. PE anti-mouse CD31 (Clone: MEC13.3, Cat#102508, RRID:AB_312915), Mouse monoclonal anti- CD34 (Clone: QBEnd/10, Cat#826401, RRID:AB_2564903) were purchased from BioLegend. Mouse monoclonal anti-PDGFRb (Clone: 42G12, Cat#ab69506, RRID:AB_1269704), Rabbit polyclonal anti-CD31 (Cat#ab28364, RRID:AB_726362), Rabbit polyclonal anti-ATF4 (Cat#ab31390) were purchased from Abcam. Rat monoclonal anti-NG2 (Clone: 546930, Cat#MA5-24247, AB_2606388), Mouse monoclonal anti-VEGF (Clone: JH121, Cat#MA5-13182, RRID:AB_10981661), Goat polyclonal anti-mouse IgG, Alexa Fluor 594 (Cat#A-11005, RRID:AB_141372), Donkey polyclonal anti-rabbit IgG, Alexa Fluor 488 (Cat#A-21206, RRID:AB_2535792), Donkey polyclonal anti-sheep IgG, Alexa Fluor 488 (Cat#A-11015, RRID:AB_2534082), Goat polyclonal anti-rat IgG, Alexa Fluor 488 (Cat#A-11006, RRID:AB_2534074), Donkey polyclonal anti-goat IgG, Alexa Fluor 488 (Cat#A-11055, RRID:AB_2534102), Goat polyclonal anti-Rabbit IgG, HRP (Cat#31460, RRID:AB_228341), Goat polyclonal anti-Mouse IgG, HRP (Cat# 31430, RRID:AB_228307) were purchased from ThermoFisher. Rat monoclonal anti-CD31 (Clone: MEC13.3, Cat#550274, RRID:AB_393571) and Mouse IgG2a, FITC (Cat#553456, RRID:AB_479604) were purchased from BD Biosciences. Sheep polyclonal anti-FAP (Cat#AF3715, RRID:AB_2102369), Mouse monoclonal CXCL12/SDF-1 (Clone: 79018, Cat#MAB-350, RRID:AB_2088149) were purchased from R&D systems. Goat anti-Collagen Type I (Cat#1310-01, RRID:AB_2753206) and Mouse IgG1 (Clone: 15H6, Cat#0102-01, RRID:AB_2793845) were purchased from Southern Biotech. Goat IgG (Cat#005-000-003, RRID:AB_2336985), Rabbit IgG (Cat#011-000-003, RRID:AB_2337118), Donkey polyclonal anti-goat IgG, HRP (Cat#705-035-147,

	RRID:AB_2313587) and Goat polyclonal anti-rabbit IgG, HRP (Cat#111-035-144, RRID:AB_2307391) were purchased from Jackson ImmunoResearch. Rabbit polyclonal anti-Ki67/MKI67 (Cat#NB500-170, RRID:AB_343263), Rabbit polyclonal anti-CD31 (Cat#NB100-2284, RRID:AB_10002513) were purchased from Novus. Antibodies were used for Western Blot, IF, IHC, ChIP, flow cytometry and multiple IF. Please refer to Supplementary Table 14 to check for their relevant application.
Validation	For most of the antibodies we validated using WT and knockout or knock down cells and different stimulations. Manufacturer's site also has validation for antibodies.

Eukaryotic cell lines

Policy information about cell lines

Cell line source(s)	B16F10 were purchased from ATCC. MH6419 (6419c5) cells are a kind gift from Ben Stanger at The University of Pennsylvania. MH6419 cells originated from the KrasLSL-G12D/wt;Trp53fl/fl;Pdx1-Cre (KPC) model of spontaneous pancreatic cancer (PMID: 29958801).
Authentication	None of the cell lines were authenticated within 1 year. The MH6419 cell line was derived from tumor developed in a genetically modified mouse and has been characterized previously (PMID: 29958801).
Mycoplasma contamination	All cell lines were tested for mycoplasma and were found negative.
Commonly misidentified lines (See ICLAC register)	The cell lines used are not listed in the ICLAC register.

Animals and other organisms

Policy information about studies involving animals; ARRIVE guidelines recommended for reporting animal research

Laboratory animals	Male and female mice 9-10 week old from the following genotypes (C57BL/6 background) were used in all in vivo experiments: Rosa26::CreERT2:Atf4wt/wt and Rosa26::CreERT2:Atf4fl/fl , Col1a1::CreERT2:Atf4wt/wt and Col1a1::CreERT2:Atf4fl/fl.
Wild animals	This study did not involve wild animals.
Field-collected samples	This study did not involve field collected samples.
Ethics oversight	All animal experiments have been approved by the University Laboratory Animal Resources (ULAR) and Institutional Animal Care and Use Committee (IACUC) of the University of Pennsylvania regulations.

Note that full information on the approval of the study protocol must also be provided in the manuscript.

Human research participants

Policy information about studies involving human research participants

Population characteristics	Characteristics for malignant melanoma with normal skin tissue array: 90 males of 54.8±12.8 years old and 86 females of 53.3±12.9 years old (Supplementary Table 18) and pancreas cancer tissue array with adjacent normal pancreas tissue: 5 males of 59.4±9.6 years old and 5 females of 61.8±6.6 years old (Supplementary Table 19). Characteristics for the formalin-fixed paraffin-embedded human melanoma tumors are not available (Fig. 3k, Supplementary Table 20) since the samples were de-identified.
Recruitment	There is no available information for the recruitment of the patients.
Ethics oversight	Ethical considerations and protocols that are used in tissue collection of the tissue arrays (malignant melanoma and pancreatic cancer) are mentioned on the Biomax webpage (https://www.biomax.us/FAQs) under the FAQ10: "All tissue is collected under the highest ethical standards with the donor being informed completely and with their consent. We make sure we follow standard medical care and protect the donors' privacy. All human tissues are collected under HIPPA approved protocols. All samples have been tested negative for HIV and Hepatitis B or their counterparts in animals and approved for commercial product development".<br>Human melanoma tumor samples were obtained from patients resected at the Hospital of the University of Pennsylvania upon signing the informed consent in accordance with the Institutional Review Board (IRB) protocol No. 703001. There is no other information available for these patients since the samples were de-identified.

Note that full information on the approval of the study protocol must also be provided in the manuscript.

Flow Cytometry

Plots

Confirm that:

☒ The axis labels state the marker and fluorochrome used (e.g. CD4-FITC).

☒ The axis scales are clearly visible. Include numbers along axes only for bottom left plot of group (a 'group' is an analysis of identical markers).

☒ All plots are contour plots with outliers or pseudocolor plots.

☒ A numerical value for number of cells or percentage (with statistics) is provided.

Methodology

Sample preparation	Primary cultures of ATF4wt/wt lung endothelial cells and single cell suspension from B16F10 tumors grown in ATF4 WT and ATF4 KO mice.
Instrument	All data acquisition was done using a FACSCanto II (BD Biosciences).
Software	BD FACSCanto II used to collect the data and FlowJo v.10 to analyze them.
Cell population abundance	Lung endothelial cells were above 80% pure post beads isolation (positive selection) and determined by flow cytometry (CD31-PE+ cells). B16F10 tumors were analyzed for live/dead cells (AmCyan).
Gating strategy	For endothelial cells: Forward scatter (FSC) versus Side scatter (SSC) gating were used for the selection of population. CD31-PE versus Count gating were used to show the positivity for CD31.<br>For tumor cells: Forward scatter (FSC) versus Side scatter (SSC) gating were used for the selection of population. FSC-A versus FSC-H gating were used for the selection of single cells only. AmCyan-A versus SSC-A gating were used to determine the live/dead cells.

☒ Tick this box to confirm that a figure exemplifying the gating strategy is provided in the Supplementary Information.

March 2021
