# Supplementary figures and images for: A stromal Integrated Stress Response activates perivascular cancer-associated fibroblasts to drive angiogenesis and tumour progression
Source: Nat Cell Biol. 2022 Jun 2;24(6):940–53. doi: 10.1038/s41556-022-00918-8 (PMC9203279; doi:10.1038/s41556-022-00918-8)

**Fig. 4g**

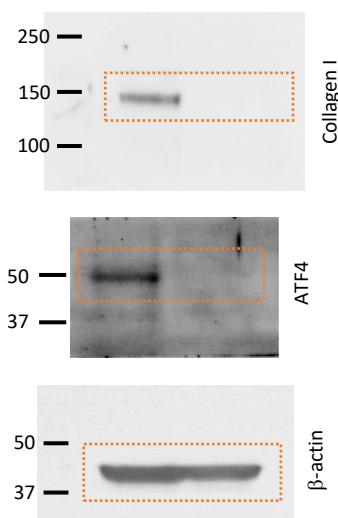

**Fig. 4j**

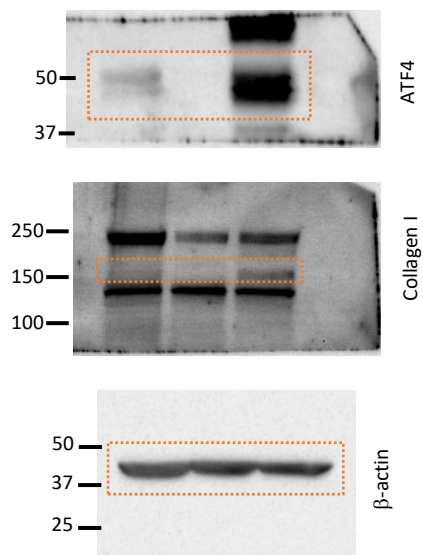

**Fig. 4k**

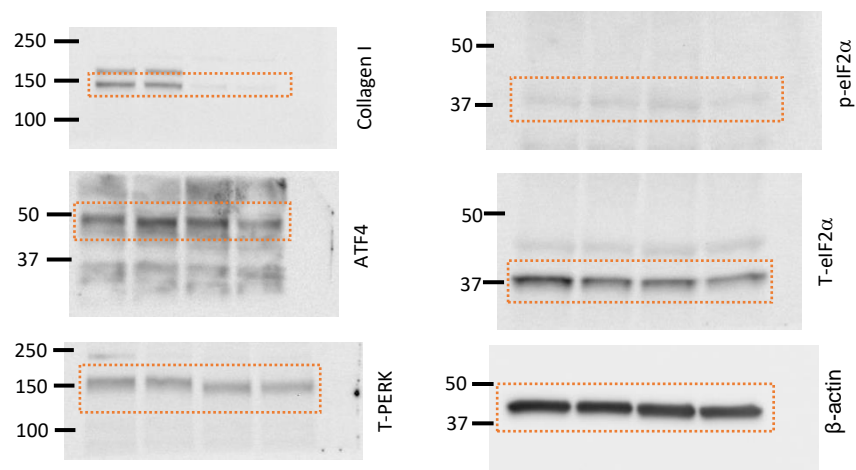

Supplement: Source Data Fig. 4 — Unprocessed western blots. [file 41556_2022_918_MOESM12_ESM.pdf]

**Fig. 5e**

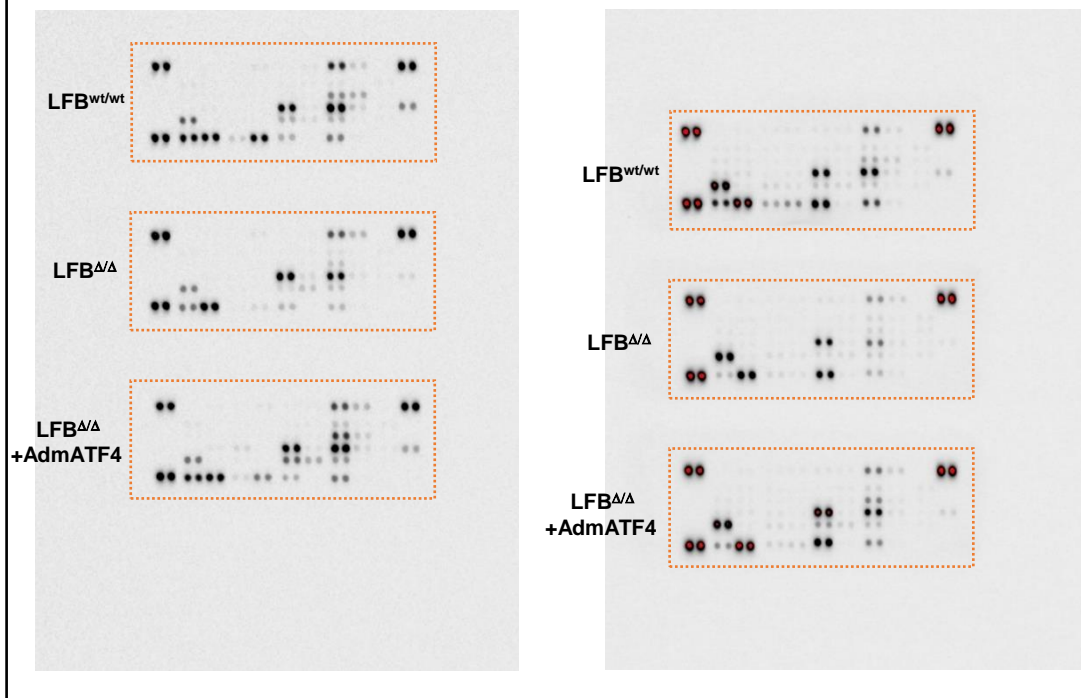

**Fig. 5g**

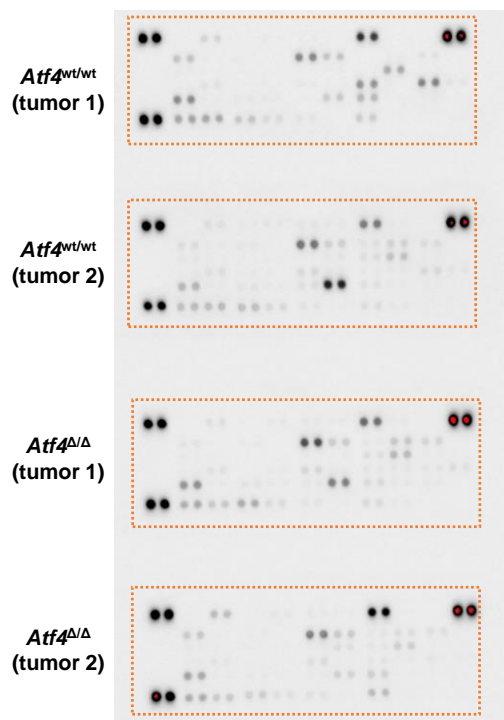

**Fig. 5l**

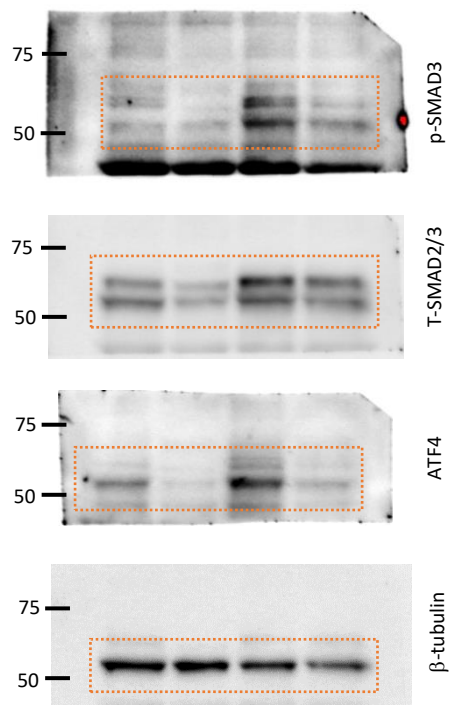

Supplement: Source Data Fig. 5 — Unprocessed western blots. [file 41556_2022_918_MOESM14_ESM.pdf]

Extended Data Fig. 7i

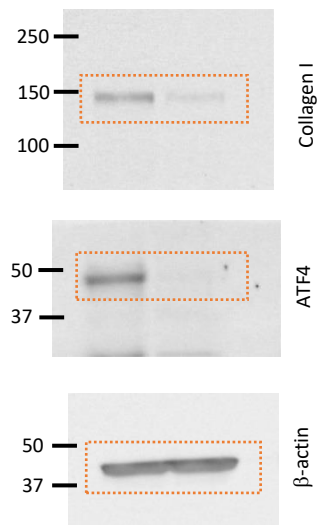

Extended Data Fig. 7l

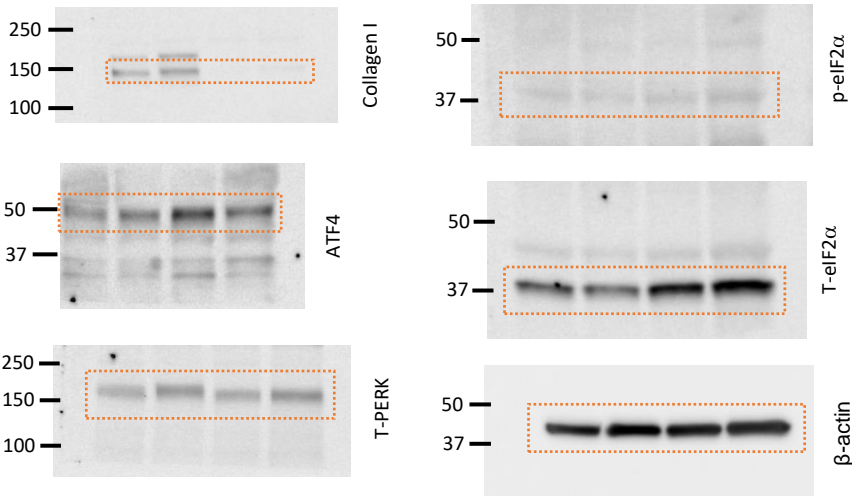

Supplement: Source Data Extended Data Fig. 7 — Unprocessed western blots. [file 41556_2022_918_MOESM23_ESM.pdf]
